# Learned lesson from COVID-19: can routine immunizations be the first line of defense against the next pandemic?

**DOI:** 10.1186/s43054-022-00105-2

**Published:** 2022-04-04

**Authors:** Antoine AbdelMassih, Hanya Gaber, Meryam El Shershaby, Maram Hanafy, Yasmin Omar, Reem Husseiny, Nada AlShehry, Habiba-Allah Ismail, Aya Kamel, Rafeef Hozaien, Ghadeer Khaled, Mohamed Amer, Aya Turki, Heba Fawzy, Stefano Puligheddu, Dalia Khaled, Nardine Nasry Thabet, Mariam Sherif Abdelaziz, Mustafa Barakat, Sara Sharaf, Ahmed Mohamed, Dina Mohsen, Amr El Feky, Hadil Adly, Eman Ibrahim, Rana Mahmoud, Mirna Reda, Felopateer Riad, Carmen Vasile, Mahitab Adel Shohdi, Nadine Hesham, Nadine El-Husseiny, Rana Ragy, Raghda Fouda

**Affiliations:** 1 Pediatric Department, Pediatric Cardiology Unit, Faculty of Medicine, Cairo University, Kasr Al Ainy Street, Cairo, 12411 Egypt; 2 Pediatric Cardio-Oncology Clinic, Children Cancer Hospital of Egypt, Cairo, Egypt; 3 Student and Internship research program (Research Accessibility Team), Faculty of Medicine, Cairo University, Cairo, Egypt; 4 Faculty of Dentistry, Cairo University, Cairo, Egypt; 5 Pixagon Graphic Design Agency, Cairo, Egypt; 6 Clinical and Chemical Pathology Department, Faculty of Medicine, Cairo University, Cairo, Egypt

**Keywords:** Trained immunity, Skewed immune response, COVID-19, Next pandemic

## Abstract

**Background:**

Single-cell sequencing studies on the lung microenvironment have revealed that the outcome of COVID-19 depends largely on the immune system response rather than the viral load. A robust innate immune response and a regulated adaptive immunity can prevent the worst outcomes such as hospitalization and the need for mechanical ventilation.

**Main body:**

Intriguingly, several vaccines pertaining to the routine vaccination schedule, not only BCG, can skew the immune response towards the aforementioned beneficial effects.

**Short conclusion:**

This means that routine immunization not only can help in the current pandemic but can also offer a rapid rescue in the subsequent epidemics or pandemics until a vaccine is developed.

## Background

COVID-19 usually manifests itself rather mildly in a flu-like fashion, however, it can manifest very aggressively through a massive, fatal cytokine cascade. It is currently established that viral load is not the first determinant of such wide variability of manifestations. The following data has led us to assume there is a solid relationship between a virally skewed innate as well as adaptive immune response and the self-induced hyper-inflammatory cytokine storm. This would shed so much light on how the virus evades the immune response as well as turn it against itself and how COVID-19 manifestations would significantly vary amongst individuals. Next-generation sequencing has helped us to understand the polarization of various limbs and components of the immune system and how such polarization can be critical to the determination of the outcomes of COVID-19 [1].

### An efficient innate immunity is critical to a balanced adaptive immune response

#### Natural killer cells

Natural killer (NK) cells represent the prime source of interferon-γ (INF-γ) during early viral infection, which together with perforin and tumor necrosis factor-ɑ (TNF-ɑ), help epitomize the cytotoxic T lymphocyte (CTL) response against the infection. A clinical trial was conducted by Hailong Guo et al. where they demonstrated different influenza viral loads on mice and their effect on both NK cells as well as CTL activity. To their surprise, reversal of defective CTL activity was achieved in mice receiving high viral loads only after effective transfer of splenic NK cells from mice with lower viral loads. This sheds more light on the close interaction between NK cells and CTL activity. In addition to that, activated NK cells speed up dendritic cell maturation via TNF-ɑ and granulocyte-monocyte colony-stimulating factor (GM-CSF). NK cells also boost the ability of CD4 cells to secrete INF-γ and lyse mycobacterium tuberculosis (TB). Moreover, NK cells exhibit T cell co-stimulatory molecules such as CD-70 and CD-86 which render them having antigen-presenting cell (APC)-like characteristics and the ability to activate T cells directly. This shows how intertwined the NK cells are with both innate as well as adaptive immune responses and how an NK cell-specific treatment will definitely enhance both limbs of the immune response [2, 3].

In parallel to the previous information, SARS COV2 produces chemokines monocyte chemoattractant protein-1 (MCP-1) and Interferon-γ-inducible protein 10 (IP-10) which in return attract NK cells to the attacked tissues, especially the lungs. Alas, the NK cell function had already been skewed to a more exhausted as well as inflammatory phenotype by the same virus. Interleukin-6 (IL-6) and IL-10, both renounced cytokines produced during severe COVID-19, play an important role in impeding the NK cell cytotoxicity. This renders the NK cells unable to express perforin, granzyme B, and INF-γ through which NK cells provide its cytotoxic degranulation of target cells as well as orchestrate both innate and adaptive immune cells. The aforementioned defective cytotoxicity prepares for further unchecked antigenic accumulation which promotes further inflammation and tissue damage aiding in a rather critical and unpromising immune response instead of a regulatory one [2].

#### Dendritic cells

Dendritic cells (DC) are bone-marrow-derived antigen-presenting cells that initiate the adaptive immune response and present antigens to T cells, thus considered a vital bridge between both adaptive and innate immune response and aiding in effective viral clearance. Etna and colleagues showed a clear difference in the dendritic cells’ phenotypes between hospitalized and asymptomatic patients. Environmental plasticity causes DC to phenotypically diversify in response to viral infections or single stimuli. They can diversify into three stable populations: P1-pDC specialized for type I IFN production, P2-pDC displaying both innate and adaptive functions, and P3-pDC specifically with adaptive functions and secreting mainly IL-6. Etna et al. proved that asymptomatic patients with COVID-19 mainly display a P1 phenotype, while on the contrary, hospitalized patients’ P2-pDC were observed in the lung micro-environment. Accordingly, DCs turn from type I IFN into TNF-α and IL-6-producing cells in patients with severe manifestations [4].

### Consequent imbalance of adaptive immune response

A lack of early and efficient innate immune response is tightly linked with a cascade of changes in the adaptive immune response characterized by an imbalance between pro-inflammatory and regulatory immune responses towards a pro-inflammatory phenotype.

#### T-helper 17 vs. T-regulatory cells

Severe SARS-COV2 has been vastly linked with massive dysregulation of various immune cells, especially in T cells and their subtypes. Additionally, disequilibrium has been noticed in T-regulatory (Treg) vs T-helper 17 (Th17) subtypes; where the former significantly decreases and the latter predominates.

Classically, Th17 cells produce pro-inflammatory cytokines (mainly IL-17, hence the name) which help recruit monocytes and neutrophils to the site of inflammation, further activating other cytokine cascades (granulocyte colony-stimulating factor G-CSF, IL-1β, IL-6, TNF-α) and chemokines (chemokine ligand 1 CXCL1, CXCL-2, CXCL10, and CCL20) (Wu D et al.). Contrarily, Treg cells are known to dampen overactive immune responses by producing anti-inflammatory cytokines such as IL-4, IL-10, and transforming growth factor-ɑ (TGF-ɑ). By doing so, it plays a crucial role in maintaining immune homeostasis.

Typically, Naïve CD4 T cells differentiate into Treg or Th17 cells under the effect of TGF-ɑ. In presence of IL-6 (in addition to TGF-ɑ), Naïve CD4 cells have been found to differentiate into the Th17 subtype. Conveniently, the IL-6 level is characteristically high in patients with severe SARS-COV2 infection, which in return inhibits TGF-β-induced Treg differentiation. Thus, by promoting a pro-inflammatory Th17 lineage and hindering Treg induction, IL-6 plays a vital role in Treg/Th17 ratio dysregulation found in severe COVID-19 infection and accordingly in the disease outcome [5, 6].

#### M1 vs. M2 macrophages and antibody responses

Monocytes and macrophages, meanwhile, are key components in the innate immune system, primarily through phagocytosis and the release of inflammatory cytokines. They exhibit various phenotypes according to their microenvironment as well as the organ they inhabit. One of the classic macrophage switches is between M1 (pro-inflammatory phenotype) and M2 (regulatory phenotype). However, Castoldi et al. highlighted that the predominance of M1 is what mainly characterizes the lung milieu in severe COVID-19, while M2 phenotype predominance indicates a much milder disease outcome. M1 macrophages harbor several chemokine and IL ligands making them highly involved in the massive inflammatory response that is the cytokine storm in severe COVID-19. While it was thought that an antibody response was required to clear SARS-CoV-2 infection, patients who produced a premature antibody response to the S protein had a higher risk of death and succumbed to the disease more quickly than those who developed antibody responses later in the disease. This surprising outcome was caused by enhanced polarization of alveolar macrophages to the pro-inflammatory M1 phenotype, which resulted in increased inflammation and impaired lung repair. This might be an antibody-dependent enhancement of disease (ADE), as shown in SARS-CoV and MERS-CoV, where early antibody production aids viral entrance into macrophages and other immune cells by allowing virion absorption through phagocytic Fc receptors [7].

#### Exhausted T cells

T cells play a cardinal role in viral clearance, where CD8 CTLs can secrete a myriad of molecules such as perforin, granzyme, and IFN-y to ensure viral clearance from the host. Simultaneously, CD4+ Th cells aid the CTLs and B cells by improving their ability to clear pathogens.

Bo Diao et al. deduced an inverse relationship between total as well as subset T cell count (CD4 and CD8) and patients’ survival in COVID-19. When monitored, cell counts were significantly lower in patients requiring intensive care unit (ICU) admission as well as patients > 60 years old compared to patients with a milder presentation and younger age, suggesting a potential cause of this cellular diminution in the elderly. In addition to that, the expression of certain exhaustion markers on T cells such as programmed cell death protein 1 (PD1) and T cell immunoglobulin mucin family member 3 (Tim3) was witnessed, denoting a synchronous decrease in T cell function as well as number the more severe the disease becomes.

Oddly enough, angiotensin-converting enzyme II (ACE-II) expression, the main SARS COV2 receptor, is normally absent on T cells. This makes us lean towards the fact that there’s another reason behind this T cellular drop in severe COVID-19 other than direct viral infection. Hence, another inverse relationship was proposed by Diao and colleagues, this time between T cell count and certain cytokine concentrations (such as IL-6, IL-10, TNF-ɑ) The aforementioned cytokines dropped significantly with disease resolution while T cell number and function resurfaced, suggesting that these cytokines may negatively regulate T cell survival. The source of the fatal cytokine storm in severe COVID-19 remains an open discussion [8].

## C-cytokines

### 1-IL-6 is it a friend or foe?

Cytokines secreted by immune cells initially serve a very important role in combating COVID-19. However, they are implicated in hyper-stimulation of the immune system that may lead to what is known as the cytokine storm, also referred to as cytokine storm syndrome (CSS), which can be ultimately deadly. At the beginning of the pandemic, it was thought that IL-6 levels are the sole determinant of the severity of COVID-19. Serum IL-6 levels were significantly lower in severe or critical COVID-19 than in other critical diseases (sepsis, cytokine release syndrome, and adult respiratory distress syndrome (ARDS) unrelated to COVID-19), according to a meta-analysis by Leisman et al, suggesting that, rather than its levels, IL-6 induction and differential signaling pathways may play a role in the severity of COVID-19 [9].

Although IL-6 has been implicated in the CSS, its role in body defense cannot be denied. As demonstrated by Mauer et al, IL-6 acts as an anti-inflammatory cytokine by promoting the M2 stage through increasing IL4 expression [10]. In addition, IL-6 induced IL-4 expression by CD4 T cells inhibits Th1 polarization and in turn, promotes Th2 response [11]. IL-6 also inhibits IFN-γ secretion by CD4 T cells, which is an essential interferon in Th1 polarization. Other processes mediated by IL-6 include regulation of IL-21, which increased B-cell IgG production, and regulation of the macrophage colony stimulation factor (M-CSF). The latter controls monocyte differentiation into macrophages [12]. IL-6 is also involved in the regulation of metabolism, maintenance of bone homeostasis, neuronal function, and regenerative processes [13].

Understanding, the differential effect of IL-6 can be achieved through a better understanding of its signaling pathways. IL-6 can issue its signals through a classic or a trans-signaling pathway. In both pathways, IL-6 binds to IL-6 receptor α (IL-6Rα) and subsequently to the signaling receptor protein gp130. In classic signaling, however, IL-6 binds to a membrane-bound form of IL-6Rα, in turn producing a regulatory response. During trans-signaling, IL-6 produces a pro-inflammatory response via binding to the soluble form of IL-6Rα which would produce a complex that would then bind to the membrane-bound gp130. Both pathways ultimately lead to activation of the Janus kinase/signal transducer and activator of the transcription pathway (JK/STAT) [14].

Reeh et al., through a data-driven computational modeling-supported systems biology approach, concluded that the pro-inflammatory vs regulatory response depends on the ratio of the IL-6 receptor α to gp130 expression independent of the JK/STAT pathway. If gp130 is expressed more, the trans-signaling pathway takes the upper hand. Most somatic cells don’t express IL-6Rα and hence don’t respond to the classic signaling pathway. Cells that express both gp130 and IL-6Rα respond to both pathways. For these cells, the ratio of IL-6α, membrane-bound IL-6Rα, soluble IL-6Rα, and gp130 would determine their response [14].

Another hypothesis to the regulatory vs. detrimental effect of IL-6 is the production of MCP-1. During early inflammation, IL-6 release is coupled with decreased production of pro-inflammatory cytokines. With the continued release of IL-6, this role is reversed and IL-6 enhances the production of MCP-1 leading to tissue damage [15].

Such findings are very important in understanding the differential effect of IL-6. Early production of IL-6 has essential beneficial viricidal effects, while its continued release and subsequent MCP-1 production can have deleterious effects on the host tissues.

### 2-IL-5 overlooked role in COVID-19

IL-5 has an eminent role in the Th2 response, particularly as an eosinophil colony-stimulating factor, promoting eosinophil survival and chemotaxis. IL-5 was also found to cause an increase in B cells and hence increasing IgM, IgA, and IgE [16].

Eosinophils have been demonstrated to have both in vivo and in vitro virucidal properties. In vitro exposure of Respiratory Syncytial Virus (RSV) to human eosinophils was found to decrease its infectivity. This was reversed by the administration of ribonuclease inhibitors. Similarly, recombinant eosinophil-derived neurotoxin causes a dose-dependent decrease in RSV infectivity. Similar findings were noted against other viruses including HIV-1, parainfluenza [17], and influenza virus [18]; has demonstrated that IL-5 transgenic mice have a higher viral clearance than wild-type control mice. Adoptive transfer of eosinophils into wild-type mice was also shown to hasten viral clearance [19].

Mechanisms by which eosinophils produce their antiviral effect include the recognition of viruses through Toll-like receptors and the production of reactive nitrogen species. (Flores-Torres et al., 2019). Eosinophils also contain preformed granules, which contain cytotoxic proteins, such as eosinophil peroxidase, major basic protein, and 2 RNases (eosinophil neurotoxin and eosinophil cationic protein) [18, 20].

During the first phase of SARS-CoV-2 infection, eosinophils have a beneficial effect by targeting viral replication. However, during the second phase of the disease, clinical symptoms start to appear due to the beginning of the immune response, and in the third stage, immunopathologies take the upper hand via a prevalent Th1 pro-inflammatory response. In the last two phases, eosinophils may contribute to such deleterious processes [21].

Accordingly, an increase in IL-5 was noted in the first two weeks following infection, followed by a plummet in the following week, and an increase again in the fourth week. This can be explained by the aforementioned role of eosinophils in viral clearance early in the disease course, but as the disease progresses, their role will not be of importance. It was suggested that IL-5, and hence eosinophil, increase in late severe cases is probably linked to immuno-pathologies, including the cytokine storm syndrome. It was suggested that this be due to eosinophil migration to the lung followed by primary lysis which would recruit phagocytes and later cause their uncontrolled activation leading to a cytokine storm. Although eosinophil increase has been noted in severe cases in various studies [22], it was not demonstrated that such increase was a direct cause of tissue damage.

In another study, Cazzaniga demonstrated that patients with absolute eosinopenia were more prone to need intensive respiratory support (49.3% vs 13.3%, *P* < .001), had higher and lower rates of hospital discharge, and had a higher mortality rate (30.6% vs 6.2%, *P* .006) compared with those who did not demonstrate absolute eosinopenia. They also had a lower hospital disgorge rate (28% vs 65.6%, *P* < .001). Absolute eosinopenia was also associated with higher C-reactive protein (CRP), and a more marked decrease in lymphocytes, monocytes, and platelet count [23]. CD8-T cell depletion and hence decrease in IL-5 production might be implicated in the noted decrease in eosinophil count in severe cases. Also, peripheral eosinopenia might be due to consumption due to high viral loads owing to its antiviral properties. This explains the unfavorable prognosis in such patients [24].

In conclusion, the role of IL-5, and in turn eosinophils, in combating SARS-CoV 2 cannot be denied. And accordingly, the upregulation of IL-5 might be helpful in improving COVID-19 outcomes.

## Main text

We can deduct from the above, that COVID-19 outcomes are dependent on the predominant response of the immune system. A strong innate immune response triggers a balanced acquired immune response with an adequate viricidal effect and sparing the host significant tissue damage; whereas a tamed innate immune response results in a pro-inflammatory acquired immune reaction with limited viricidal response and significant host tissue damage.

There is strong evidence that some vaccines pertaining to routine immunization schedule have non-specific effects on the immune system that can skew the immunity of the host towards a more beneficial response to COVID-19. To our knowledge, Chumakov and colleagues were the first to outline the role of old vaccines in improving innate immune response against COVID-19, however they did not specify the individual effect of such vaccines on the innate or adaptive immunity and how these changes can match the changes unleashed by single sequencing of the lung micro-environment [25].

We thereby hypothesize, that adherence to routine immunization or even reviving their effects by boosters in the old aged can have a positive impact on the outcomes of COVID-19 and can aid the ongoing efforts of implementation of COVID-19 vaccines.

### Examples of non-specific effects of non-COVID-19 vaccines

#### Bacillus Calmette–Guérin—tuberculosis

The Bacillus Calmette–Guérin (BCG) vaccine has cross-protective effects on diseases other than BCG. Trained immunity, characterized by temporary epigenetic reprogramming of macrophages, explains this effect. It leads to increased inflammatory cytokine production and consequently a potent immune response. Early in the immune response process, the source of IL-6 is primarily from innate immune cells. When its activity as a pro-inflammatory cytokine persists, the acute beneficial inflammation turns deleterious through continuous MCP-1 secretion. Freyne et al. observed that infants who had received the BCG vaccine had increased production of IL-6 and decreased MCP-1. So even though BCG increases the main cytokine involved in COVID-19 complications, it blocks its detrimental effects through inhibiting the production of MCP-1 [26].

#### Measles, mumps, rubella

Vaccination with measles seems to induce a transient suppression of lymphocyte proliferation but a slight increase in the innate immune responses. An animal model revealed that as a reaction to the living measles virus, CD4-T cells undergo more selective maturation to Th2 than to Th1. Interestingly, another study on several young adults conducted in Colchester showed that cellular immunity lasts for a long time after receiving two doses of the measles, mumps, rubella (MMR) vaccine and 7.4% of the study population showed a skewed immune response towards Th2 [27].

#### Diphtheria, tetanus, pertussis

White et al. analyzed T cell responses to diphtheria, tetanus, acellular pertussis (DTaP vaccine). They found a pattern of Th-2 mediated cytokine production, consistent with data of TT-specific IgE antibodies. There was also an increased Tetanus Toxoid -specific IL-5 response in the DTaP-R group and a weak association with the pro-inflammatory IFN-γ. Thus, there seems to be a correlation between preexisting Th2-polarized cellular immune memory and the DTaP vaccine. It is consistent with a regulatory rather than pro-inflammatory immune response in patients vaccinated with the Dtap vaccine. DTaP vaccine antigens administered 2-3 years after priming produces a Th-2 preference, along with IgE production and subsequent increase in IL5 and eosinophil recruitment [28].

#### Oral polio vaccine

Oral polio vaccine (OPV) was associated with a lower rate of overall hospital admission with other non-polio-related infections and significantly lower rates of lower respiratory infections. The principal way OPV confers immunity is through activation of NK Cells. OPV inhibits the CD155 receptor, thereby eliminating its immuno-inhibitory effect on NK cells and T cells [29].

Table 1 summarizes the above effect of vaccinations.

**Table 1:** Example of routine immunizations and their non-specific effects on the immune system towards a beneficial response against COVID-19

Vaccine	Reference (number in the text)	Non-specific beneficial effects in the context of COVID-19
BCG	Freyne et al. [26]	Increases IL-6 and blocks its deleterious effects through inhibiting MCP-1
MMR	Pauksen et al. [27]	Skew adaptive immunity towards Th2 response
DtaP	White et al. [28]	Skew adaptive immunity towards Th2 response Increases Ig E antibodies and increases IL-5
OPV	Kučan Brlić et al. [29]	Improves innate immunity through potentiating NK cells through reduction of CD155 receptor

*Abbreviations*: *BCG* Bacillus Calmette–Guérin, *COVID-19* coronavirus disease 2019, *DTaP* diphtheria, tetanus, acellular pertussis, *CD* cluster of differentiation, *Ig* immunoglobulin, *IL* interleukin, *MCP* monocyte chemotactic protein, *MMR* measles, mumps, rubella, *OPV* oral polio vaccine, *Th* Thelper

### Implications of such a hypothesis

#### Unequal distribution of cases around the world

As of 9 August 2021, a total of 4,033,274,676 vaccine doses have been administered around the world. Examination of the confirmed cases and death rates across the globe clearly shows a substantial variation in incidence and mortality between Western and Eastern countries. Countries affected early on, such as China and South Korea, have recovered quickly with relatively positive statistics (in China, only about 65 cases/mln and three deaths/mln; South Korea, about 4000 cases/mln and 41 deaths/mln). Other Southeast Asian countries have also maintained rather low statistics. For example, countries like Vietnam (1867 cases per million and only about 24 deaths per million), Thailand (< 10,000 cases/mln, < 80 deaths/mln), Laos (< 1000 cases/mln and < 1 death/mln). In contrast, Western countries had a much higher rate of infection and mortality. At the present moment, the USA reports more than 100,000 cases/mln and more than 1800 deaths/mln; the UK > 87,000 cases/mln and > 1,900 deaths/mln. Multiple theories attempt to explain this difference in the development of the pandemic in countries of the eastern and the western hemispheres. One of the most accepted theories is that the lack of testing and economic resources in these countries causes underreporting. Another hypothesis is the impact of temperature on cases’ distribution and mortality [30].

We propose a different factor for this unequal distribution; the widespread coverage of routine immunizations in developing countries over developed countries. A study showed that vaccine hesitancy increases in countries with higher gross domestic product. The latter finding might be due to increased access to social media. In the US and Canada, only 72% believe vaccines are safe. In North Europe from Ireland and the UK through the Nordic countries, the figure is about 73%. In other parts of Europe, confidence in vaccines is even lower. In Germany, France, Austria, Switzerland, and the Benelux countries, an average of 59% believe vaccines are safe. In eastern Europe, the figure is 50%. However, poorer regions have higher percentages. In South Asia, 95% of people think vaccines are safe. In east Africa, 92%. In Bangladesh, vaccination campaigns have contributed to a fall in childhood mortality. Rwanda has lifted its national immunization rate from 30% in 1995 to 95% today [31].

Several reasons justify the lack of vaccination coverage in western countries. Wealthier countries are more connected to the internet and social media and have more access to opposing views. However, some of the most notorious spikes in anti-vaccination sentiment predate the rise of social media. A now-discredited article appeared in 1998 in The Lancet by a group of doctors led by Andrew Wakefield, who linked the MMR vaccine to autism. It led to a crisis of confidence among some parents, an echo of which persists today. Opposition to vaccination often appears among people with similar beliefs and a particular worldview. And so they resist the advice of doctors or governments. In New York, there have been measles outbreaks among vaccine-opposing groups of strictly Orthodox Jews. Some non-religious parents persuade themselves and each other that the MMR vaccine can overpower a child's immune system and cause autism. Despite large-scale studies in countries such as Denmark finding no link between MMR and autism [31].

#### Why children might be at a lower risk of complications of COVID-19? And should we give boosters of the routine immunizations to the old aged to boost their non-specific protective effects?

It is critical to understand why the course of COVID-19 is affecting different groups of individuals with varying severity during the ongoing pandemic. Immune-modulation and “inflammaging” is the most accepted theory. Old-aged individuals have an impaired innate immune response, and a higher Th1 mediated immune response. This pro-inflammatory tendency is not fully understood. One of the proposed mechanisms is the loss of routine immunization responses with age. As mentioned earlier, routine immunization strengthens innate immunity and redirects adaptive immunity towards a regulatory Th2 response. The specific effects and non-specific effects of vaccines wane with age [32].

The lowest vaccine effectiveness for influenza vaccine is associated with age 50 years and older. Vaccine-induced protection decreases due to more fragility with advancing age. However, the average rate of disease reduction in those over 50 is similar to other age groups. Due to the indirect effect of herd immunity [33].

According to a study on Vaccine Efficacy (VE) estimates by age, the highest point estimates were in children aged 9–17 years (58%; 95% CI, 27–76), and the lowest were in adults aged 18–49 years (44%; 95% CI, 21–60) and adults aged ≥ 65 years (43%; 95% CI, − 18 to 72). In another study, VE ranged from 69% (95% CI, 56%–77%) in children aged six months–8 years to 38% (95% CI, − 16% to 67%) in adults aged ≥ 65 years [34].

A third study showed a similar decline. 44% (95% CI: -11 to 72) for those younger than 65 years and 19% (95% CI: -146 to 73) for those 65 or older. In the first 100 days after vaccination, VE was 61% (95% CI: 5 to 84), 42% (95% CI: -39 to 75) in the following 20 days, and zero after that. This decline mainly affected people aged 65 or over [35].

The impaired vaccination responses in the elderly are due to changes in the number and quality of the T cell compartments with age. The balance shifts so that T cells prefer short-lived effector responses over memory or T follicular helper cells (Tfh) responses. Vaccine-induced antibodies consistently have a reduced protective capacity. Multiple targetable alterations in T cells have been identified as contributing to these age-related deficits [36].

When compared to young adults, older people produce fewer antibodies. By 28 days, vaccine-specific antibody levels were comparable across age groups, and viremia was under control. The aging immune system can generate adequate initial responses but at a slower rate. Most vaccines are administered to older people to increase preexisting immunity, making it difficult to investigate these reactions. However, some vaccines were studied. Yellow fever vaccination response wanes over years from childhood to adults above the age of fifty. This leads to less effector B cell response in old aged individuals compared to children. Long-term survival of yellow fever YF-specific CD4 T cells decreased with age, indicating poor immunologic memory formation for CD4 T cells. Older people had delayed and lower primary antibody responses to hepatitis B and Japanese encephalitis virus (JEV). In JEV, over half of those over 60 did not attain the antibody levels necessary for a protective response, compared to fewer than 15% of young people [15]. IFN-γ, a major effector cytokine, and IL-10 production were substantially lower in the older cohort compared to the younger cohort [36].

Immune cell senescence caused by telomere shortening might result in these poor vaccination responses. Age-related changes in T cells and B cells may also account for these changes in the elderly. Primary vaccination responses in the elderly show characteristics associated with diminished effector T-cell growth, altered effector functioning, and decreased long-term immunologic memory formation [37].

In conclusion, vaccination responses decrease with age. And “inflammaging” might partly be due to the loss of the trained immunity effects of routine immunizations with age. Such findings might support giving the old aged individuals, boosters of the aforementioned vaccines to improve their immune responses against COVID-19.

**Fig. 1:**
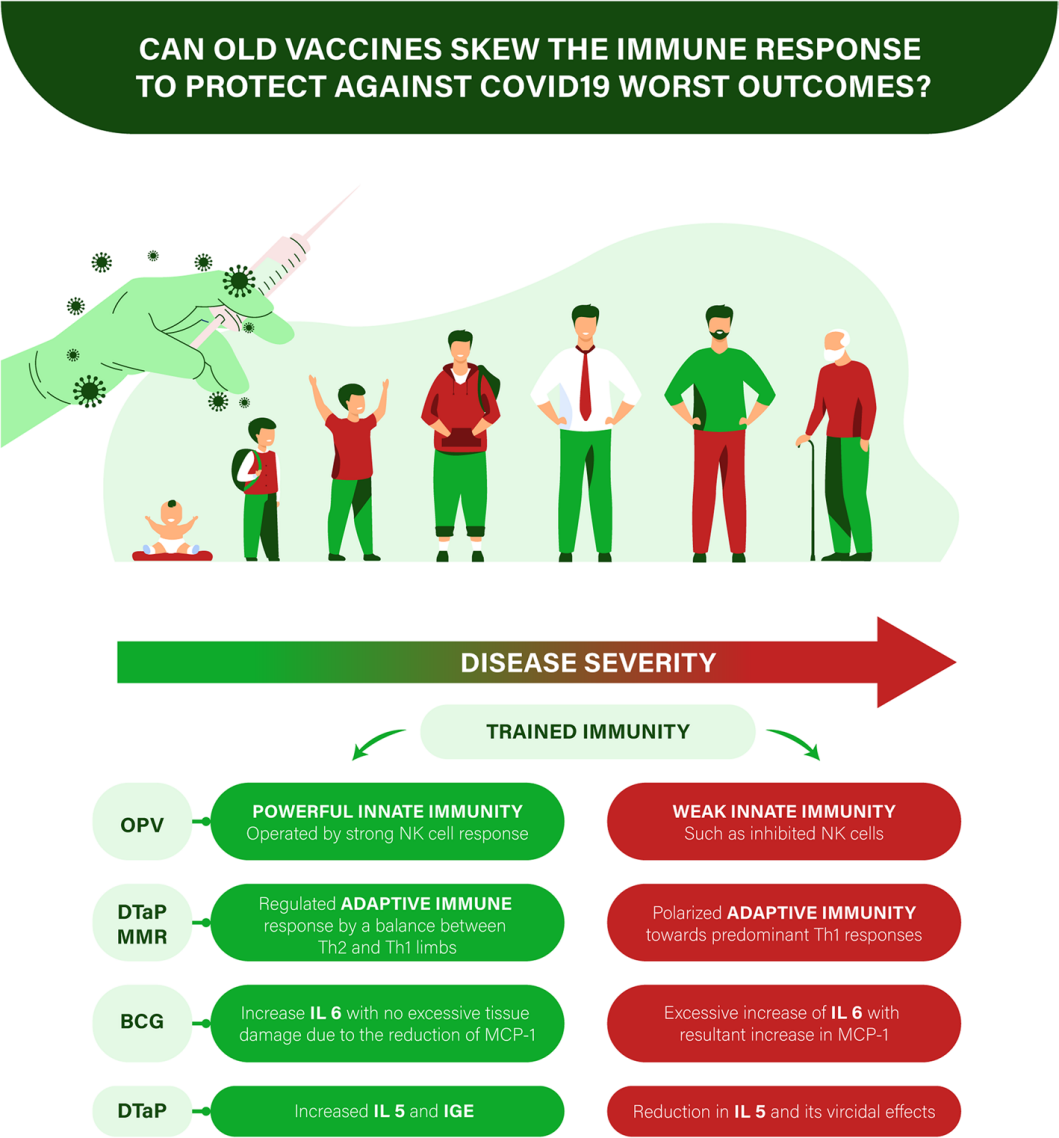
Can Old vaccines skew the immune response to protect against COVID-19 worst outcomes? Abbreviations: BCG, Bacillus Calmette–Guérin; COVID-19, coronavirus disease 2019; DTaP, diphtheria, tetanus, acellular pertussis; CD, cluster of differentiation; Ig, immunoglobulin; IL, interleukin; MCP, monocyte chemotactic protein; MMR, measles mumps rubella; OPV, oral polio vaccine; Th, T helper

Figure 1 summarizes all the above hypotheses.

## Conclusion

It is obvious from the above that several old vaccines can skew the immune system responses, potentiating the innate immunity and providing a balance between Th1 and Th2 responses of the adaptive immunity; thus, preventing significant tissue damage resulting from the immune system reactivity to SARS-CoV-2. This overlooked role of routine immunization can offer an additional role in the fight of the current pandemic, alongside with the newly developed COVID-19 vaccines. It can also provide a rapid rescue to the next pandemics, until new vaccines are developed.

## Data Availability

Not applicable

## Abbreviations

ACE: Angiotensin-converting enzyme
APC: Antigen-presenting cells
BCG: Bacillus Calmette–Guérin
CD: Cluster of differentiation
CI: Confidence interval
COVID-19: Coronavirus disease 2019
CTL: Cytotoxic T lymphocytes
CX/CC: Chemokine
DC: Dendritic cells
HIV: Human immunodeficiency virus
IL: Interleukin
INF: Interferon
JEV: Japanese encephalitis virus
JK: Janus kinase
MCP: Monocyte chemotactic protein
MMR: Measles, mumps, rubella
NK: Natural killer cells
PD: Programmed death
RNA: Ribonucleic acid
RSV: Respiratory syncitial virus
SARS-CoV-2: Severe acute respiratory virus coronavirus
TGF: Transforming growth factor
Th: T helper
TNF: Tumor necrosis factor
Treg: T regulatory cells
TT: Tetanus toxoid
UK: United Kingdom
YF: Yellow fever

## References

[1] Mortaz E, Tabarsi P, Varahram M et al (2020) The Immune Response and Immunopathology of COVID-19. Front Immunol 11:2037 10.3389/fimmu.2020.02037 PMC7479965 32983152

[2] Ahmed F, Jo D-H, Lee S-H (2020) Can Natural Killer Cells Be a Principal Player in Anti-SARS-CoV-2 Immunity? Front Immunol 11. 10.3389/fimmu.2020.586765. Epub ahead of print 10.3389/fimmu.2020.586765 PMC7750385 33365027

[3] Guo H, Kumar P, Malarkannan S (2011). Evasion of natural killer cells by influenza virus. J Leukoc Biol.

[4] Etna MP, Signorazzi A, Ricci D et al (2021) Human plasmacytoid dendritic cells at the crossroad of type I interferon-regulated B cell differentiation and antiviral response to tick-borne encephalitis virus. PLOS Pathog 17:e1009505 10.1371/journal.ppat.1009505 PMC8078780 33857267

[5] Sakaguchi S, Yamaguchi T, Nomura T et al (2008) Regulatory T Cells and Immune Tolerance. Cell 133:775–87 10.1016/j.cell.2008.05.009 18510923

[6] AbdelMassih A, Yacoub E, Husseiny RJ et al (2021) Hypoxia-inducible factor (HIF): The link between obesity and COVID-19. Obes Med 22:100317 10.1016/j.obmed.2020.100317 PMC7832240 33521378

[7] Kosyreva A, Dzhalilova D, Lokhonina A et al (2021) The Role of Macrophages in the Pathogenesis of SARS-CoV-2-Associated Acute Respiratory Distress Syndrome. Front Immunol 12:682871 10.3389/fimmu.2021.682871 PMC8141811 34040616

[8] Diao B, Wang C, Tan Y et al (2020) Reduction and Functional Exhaustion of T Cells in Patients With Coronavirus Disease 2019 (COVID-19). Front Immunol 11. 10.3389/fimmu.2020.00827. Epub ahead of print 10.3389/fimmu.2020.00827 PMC7205903 32425950

[9] Leisman DE, Ronner L, Pinotti R (2020). Cytokine elevation in severe and critical COVID-19: a rapid systematic review, meta-analysis, and comparison with other inflammatory syndromes. Lancet Respir Med.

[10] Mauer J, Chaurasia B, Goldau J (2014). Signaling by IL-6 promotes alternative activation of macrophages to limit endotoxemia and obesity-associated resistance to insulin. Nat Immunol.

[11] Diehl S, Rincón M (2002). The two faces of IL-6 on Th1/Th2 differentiation. Mol Immunol.

[12] Chomarat P, Banchereau J, Davoust J (2000). IL-6 switches the differentiation of monocytes from dendritic cells to macrophages. Nat Immunol.

[13] Scheller J, Chalaris A, Schmidt-Arras D (2011). The pro- and anti-inflammatory properties of the cytokine interleukin-6. Biochim Biophys Acta - Mol Cell Res.

[14] Reeh H, Rudolph N, Billing U et al (2019) Response to IL-6 trans- and IL-6 classic signalling is determined by the ratio of the IL-6 receptor α to gp130 expression: fusing experimental insights and dynamic modelling. Cell Commun Signal 17:46 10.1186/s12964-019-0356-0 PMC6525395 31101051

[15] Velazquez-Salinas L, Verdugo-Rodriguez A, Rodriguez LL et al (2019) The Role of Interleukin 6 During Viral Infections. Front Microbiol 10. 10.3389/fmicb.2019.01057. Epub ahead of print 10.3389/fmicb.2019.01057 PMC6524401 31134045

[16] Kouro T, Takatsu K (2009). IL-5- and eosinophil-mediated inflammation: from discovery to therapy. Int Immunol.

[17] Linch SN (2012). Interleukin 5 is protective during sepsis in an eosinophil-independent manner. Am J Respir Crit Care Med.

[18] Ramirez GA, Yacoub M-R, Ripa M (2018). Eosinophils from Physiology to Disease: A Comprehensive Review. Biomed Res Int.

[19] Stevens RL (2007) Viral infections: beneficial role of eosinophils. Blood 110:1406

[20] Flores-Torres AS, Salinas-Carmona MC, Salinas E (2019). Eosinophils and Respiratory Viruses. Viral Immunol.

[21] Siddiqi HK, Mehra MR (2020). COVID-19 illness in native and immunosuppressed states: A clinical–therapeutic staging proposal. J Hear Lung Transplant.

[22] Luecke E, Jeron A, Kroeger A (2021). Eosinophilic pulmonary vasculitis as a manifestation of the hyperinflammatory phase of COVID-19. J Allergy Clin Immunol.

[23] Cazzaniga M, Fumagalli LAM, D’angelo L et al (2021) Eosinopenia is a reliable marker of severe disease and unfavourable outcome in patients with COVID‐19 pneumonia. Int J Clin Pract 75. 10.1111/ijcp.14047. Epub ahead of print. 10.1111/ijcp.14047 PMC7995195 33497517

[24] Azkur AK, Akdis M, Azkur D (2020). Immune response to SARS-CoV-2 and mechanisms of immunopathological changes in COVID-19. Allergy.

[25] Chumakov K, Avidan MS, Benn CS et al (2021) Old vaccines for new infections: Exploiting innate immunity to control COVID-19 and prevent future pandemics. Proc Natl Acad Sci 118. 10.1073/pnas.2101718118. Epub ahead of print 10.1073/pnas.2101718118 PMC8166166 34006644

[26] Freyne B, Donath S, Germano S (2018). Neonatal BCG Vaccination Influences Cytokine Responses to Toll-like Receptor Ligands and Heterologous Antigens. J Infect Dis.

[27] Pauksen K, Sjölin J, Linde A (1997). Th1 and Th2 cytokine responses after measles antigen stimulation in vitro in bone marrow transplant patients: response to measles vaccination. Bone Marrow Transplant.

[28] White OJ, Rowe J, Richmond P (2010). Th2-polarisation of cellular immune memory to neonatal pertussis vaccination. Vaccine.

[29] Kučan Brlić P, Lenac Roviš T, Cinamon G (2019). Targeting PVR (CD155) and its receptors in anti-tumor therapy. Cell Mol Immunol.

[30] Fakhry AbdelMassih A, Ghaly R, Amin A (2020). Obese communities among the best predictors of COVID-19-related deaths. Cardiovasc Endocrinol Metab.

[31] Hussain A, Ali S, Ahmed M et al (2018) The Anti-vaccination Movement: A Regression in Modern Medicine. Cureus. 10.7759/cureus.2919. Epub ahead of print 10.7759/cureus.2919 PMC6122668 30186724

[32] Anuradha B, Santosh C, Hari Sai Priya V et al (2007) Age-related waning of in vitroInterferon-γ levels against r32kDaBCG in BCG vaccinated children. J Immune Based Ther Vaccines 5:8 10.1186/1476-8518-5-8 PMC1899498 17555578

[33] Meng Z (2020). Immunogenicity of influenza vaccine in elderly people: a systematic review and meta-analysis of randomized controlled trials, and its association with real-world effectiveness. Human Vaccines Immunotherapeut.

[34] Treanor JJ, Talbot HK, Ohmit SE (2012). Effectiveness of seasonal influenza vaccines in the United States during a season with circulation of all three vaccine strains. Clin Infect Dis.

[35] Castilla J, Martínez-Baz I, Martínez-Artola V et al (2013) Decline in influenza vaccine effectiveness with time after vaccination, Navarre, Spain, season 2011/12. Euro Surveill 18. 10.2807/ese.18.05.20388-en. Epub ahead of print 10.2807/ese.18.05.20388-en 23399423

[36] Savino W (2006) The thymus is a common target organ in infectious diseases. PLoS Pathog 2:e62 10.1371/journal.ppat.0020062 PMC1483230 16846255

[37] Najarro K, Nguyen H, Chen G (2015). Telomere Length as an Indicator of the Robustness of B- and T-Cell Response to Influenza in Older Adults. J Infect Dis.

